# Aging of the skeletal muscle extracellular matrix drives a stem cell fibrogenic conversion

**DOI:** 10.1111/acel.12578

**Published:** 2017-03-30

**Authors:** Kristen M. Stearns‐Reider, Antonio D'Amore, Kevin Beezhold, Benjamin Rothrauff, Loredana Cavalli, William R. Wagner, David A. Vorp, Alkiviadis Tsamis, Sunita Shinde, Changqing Zhang, Aaron Barchowsky, Thomas A. Rando, Rocky S. Tuan, Fabrisia Ambrosio

**Affiliations:** ^1^ Department of Physical Medicine and RehabilitationUniversity of PittsburghKaufmann Medical Building, Suite 201, 3471 Fifth AvenuePittsburghPA15213USA; ^2^ McGowan Institute for Regenerative MedicineUniversity of Pittsburgh450 Technology Drive, Suite 300PittsburghPA15219USA; ^3^ Department of SurgeryUniversity of Pittsburgh450 Technology Drive, Suite 300PittsburghPA15219USA; ^4^ Department of Environmental and Occupational HealthUniversity of Pittsburgh100 Technology Drive, Suite 328PittsburghPA15219USA; ^5^ Center for Cellular and Molecular EngineeringDepartment of Orthopaedic SurgeryUniversity of Pittsburgh450 Technology Drive, Bridgeside Point II, Suite 221PittsburghPA15219USA; ^6^ Center for Vascular Remodeling and RegenerationCenter for Bioengineering (CNBIO)University of Pittsburgh300 Technology Drive, Suite 300PittsburghPA15219USA; ^7^ Department of BioengineeringUniversity of Pittsburgh213 Center for Bioengineering, 300 Technology DrivePittsburghPA15219USA; ^8^ Department of EngineeringUniversity of Leicester127 Michael Atiyah Building, University RoadLeicesterLE1 7RHUK; ^9^ Glenn Center for the Biology of Aging and Department of Neurology and Neurological SciencesStanford University School of MedicineStanfordCA94305USA; ^10^ RR&D CenterVA Palo Alto Health Care SystemPalo AltoCA94304USA

**Keywords:** aging, extracellular matrix, satellite cells, muscle stem cells, skeletal muscle

## Abstract

Age‐related declines in skeletal muscle regeneration have been attributed to muscle stem cell (MuSC) dysfunction. Aged MuSCs display a fibrogenic conversion, leading to fibrosis and impaired recovery after injury. Although studies have demonstrated the influence of *in vitro* substrate characteristics on stem cell fate, whether and how aging of the extracellular matrix (ECM) affects stem cell behavior has not been investigated. Here, we investigated the direct effect of the aged muscle ECM on MuSC lineage specification. Quantification of ECM topology and muscle mechanical properties reveals decreased collagen tortuosity and muscle stiffening with increasing age. Age‐related ECM alterations directly disrupt MuSC responses, and MuSCs seeded *ex vivo* onto decellularized ECM constructs derived from aged muscle display increased expression of fibrogenic markers and decreased myogenicity, compared to MuSCs seeded onto young ECM. This fibrogenic conversion is recapitulated *in vitro* when MuSCs are seeded directly onto matrices elaborated by aged fibroblasts. When compared to young fibroblasts, fibroblasts isolated from aged muscle display increased nuclear levels of the mechanosensors, Yes‐associated protein (YAP)/transcriptional coactivator with PDZ‐binding motif (TAZ), consistent with exposure to a stiff microenvironment *in vivo*. Accordingly, preconditioning of young fibroblasts by seeding them onto a substrate engineered to mimic the stiffness of aged muscle increases YAP/TAZ nuclear translocation and promotes secretion of a matrix that favors MuSC fibrogenesis. The findings here suggest that an age‐related increase in muscle stiffness drives YAP/TAZ‐mediated pathogenic expression of matricellular proteins by fibroblasts, ultimately disrupting MuSC fate.

## Introduction

The ability of skeletal muscle to repair after injury is largely dependent on muscle stem cells (MuSCs; or ‘satellite cells’; Mauro, 1961). Following injury in young, healthy muscle, MuSCs activate, proliferate, and fuse to form myofibers (Carlson & Faulkner, 1983; Bonilla *et al*., 1988). However, in the case of aged muscle, the regenerative cascade is characterized by a shift from functional myofiber repair toward increased extracellular matrix (ECM) deposition (Carlson, 1995; Grounds, 1998). This impaired regeneration, among other things, appears to be attributed to dysfunction in MuSC proliferative capacity (Conboy *et al*., 2003) and MuSC divergence toward a fibrogenic lineage (Brack *et al*., 2007).

The skeletal muscle ECM has long been recognized as playing an important role in the structural features of the tissue. The ECM provides a framework for the transmission of force and contributes to the elastic tissue response. More recently, the ECM has been shown to play a dynamic role in directing resident cell function. Microenvironmental alterations, including matrix disruption, altered skeletal muscle vascularity, and fibrosis, often precede and/or accompany declines in the maintenance of skeletal muscle mass and regenerative potential (Croisier, 2004; Gargioli *et al*., 2008). Repetitive injuries that result in fibrotic deposition are associated with increased likelihood of secondary injuries and an impaired healing capacity (Croisier, 2004).

Skeletal muscle regenerative capacity is reliant on a dynamic interplay between MuSCs and the microenvironment, or niche. The myofiber basal lamina, or endomysium, is comprised of an ECM network that is in direct contact with MuSCs. *in vitro* systems have confirmed the exquisite sensitivity of stem cells to extrinsic mechanical and structural cues emanating from the surrounding microenvironment (Engler *et al*., 2006; Gilbert *et al*., 2010). *In vivo* studies investigating modulation of the microenvironment have similarly demonstrated effects on the proliferation, migration, and myogenicity of both endogenous (Conboy *et al*., 2005; Brack *et al*., 2007) and transplanted MuSCs (Palermo *et al*., 2005; Gargioli *et al*., 2008; Ambrosio *et al*., 2009, 2010; Distefano *et al*., 2013), effects that ultimately affect muscle regeneration and function (Ambrosio *et al*., 2009; Distefano *et al*., 2013). Still, little is known about how aging affects properties of the skeletal muscle ECM and how such alterations may affect MuSC fate.

In this report, we quantify age‐related alterations in the skeletal muscle ECM and demonstrate that aging is associated with pathogenic ECM architecture and increased muscle stiffness. These age‐related alterations affect stem cell behavior, and both the aged ECM and matricellular proteins secreted by aged fibroblasts drive MuSC differentiation toward a fibrogenic lineage. Aged fibroblasts display an increased nuclear translocation of Yes‐associated protein (YAP)/transcriptional coactivator with PDZ‐binding motif (TAZ), mechanotransductive transcriptional regulators that are activated in response to a stiff microenvironment (Dupont *et al*., 2011). Accordingly, seeding young fibroblasts onto a substrate engineered to mimic the stiffness of aged muscle drives YAP/TAZ nuclear translocation and promotes a secretory profile that favors MuSC fibrogenesis at the expense of myogenicity. Further supporting a role for YAP/TAZ in regulating matricellular protein expression by fibroblasts, we demonstrate that pharmacologic inhibition of YAP/TAZ nuclear translocation in aged fibroblasts drives elaboration of a matrix that is myogenically favorable for MuSCs. These results suggest that age‐related alterations in the ECM induce a pathogenic fibroblast phenotype via YAP/TAZ signaling to ultimately disrupt MuSC fate. These findings may provide insight into the predisposition for fibrosis formation following injury of aged muscle.

## Results

### Aged skeletal muscle displays a decreased collagen fibril tortuosity and increased stiffness in the direction of collagen alignment

To investigate the effect of aging on biophysical aspects of the ECM, we performed second harmonic generation (SHG) imaging of intact gastrocnemius muscle from young (3–4 months old) and old (22–24 months old) male C57BL/6 mice. From the SHG images, the collagen fibril orientation and tortuosity were analyzed using a customized algorithm (Takanari *et al*., 2013; Koch *et al*., 2014). Structural alterations in collagen fiber alignment (Courtney *et al*., 2006) and/or tortuosity (Stella *et al*., 2010b) affect the ECM degree of mechanical anisotropy and can provide important insight into the biologic basis for age‐related alterations in ECM mechanical properties.

**Figure 1:**
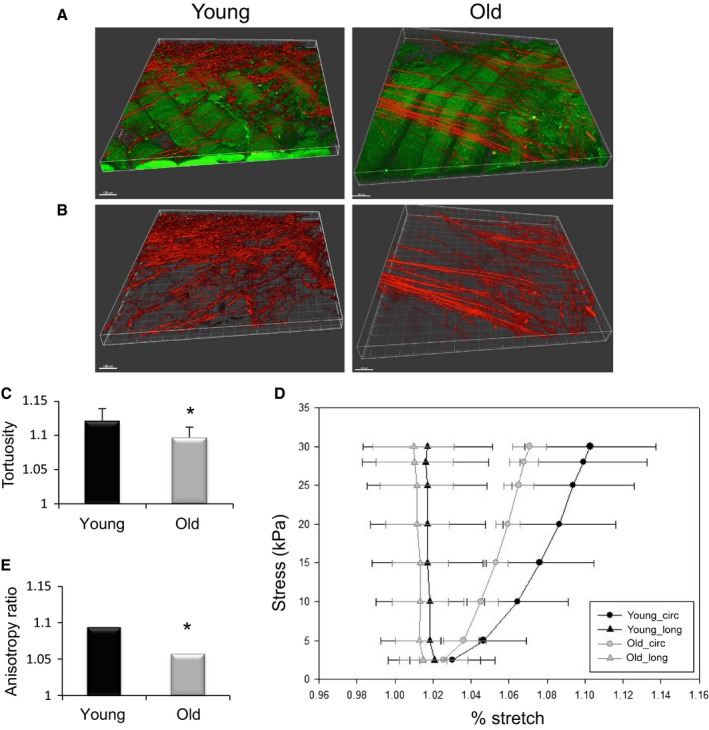
Collagen fiber orientation, tortuosity, and mechanical properties of young and old skeletal muscle. (A) Representative images from young and old skeletal muscle from multiphoton imaging and second harmonic generation (red = collagen, green = muscle fibers). For image processing, only the red channel was selected (B). While there was no significant difference in collagen fiber orientation between old and young samples, collagen fibers in old skeletal muscle were significantly less tortuous than those in young. (*P = 0.04; *n* = 6/group) (C). Biaxial mechanical testing indicated a reduction in compliance in the circumferential direction in old skeletal muscle, as compared to young, with increasing stress (*n* = 7/group) (D). Muscle from old mice displays a significantly lower anisotropy ratio (AR) (AR = (λXD−1)/(λPD−1); PD = preferred fiber direction; XD = cross‐preferred fiber direction) as compared to that from young mice (*P = 0.017) (E). Data are displayed as mean ± SD. Bar = 100 μm.

We observed no significant difference in the collagen fiber orientation between young and old animals, and, regardless of age, the majority of the fibers were aligned perpendicular to the direction of the myofibers (Fig 1A; *P* = 0.33). These findings are consistent with previous reports using picrosirius red staining (Wood *et al*., 2014). In contrast, there was a significantly decreased collagen fiber tortuosity in the ECM of old muscle, as compared to that observed in young ECM (Fig 1C; *P* = 0.04).

We next quantified two‐dimensional mechanics with biaxial testing of intact gastrocnemius muscles obtained from young and old mice. While uniaxial tensile testing provides information regarding overall tissue stiffness, only a two‐dimensional testing modality, such as biaxial testing, is capable of assessing the impact of structural reorganization on organ‐level mechanics (Stella *et al*., 2010a). In addition, such testing allows for calculation of the anisotropy ratio, which provides a standard metric for macroscopic tissue deformation (Courtney *et al*., 2006). Finally, the biaxial testing and sample preparation protocol eliminates the mechanical contribution of the tendon, which may confound results (Kragstrup *et al*., 2011). Calculation of the anisotrophy ratio following biaxial testing revealed a decreased degree of anisotropy in aged skeletal muscle. This decreased anisotropy was consistent with an increased stiffness in the circumferential, but not longitudinal, direction (Fig 1D,E), when compared to young counterparts.

In accordance with the increased stiffness of aged muscle, histological analysis of aged decellularized muscle also revealed a decreased abundance of the highly compliant ECM components, elastin and collagen type III, when compared to young counterparts (Fig S1, Supporting information).

### Aging promotes a pathogenic fibroblast phenotype

**Figure 2:**
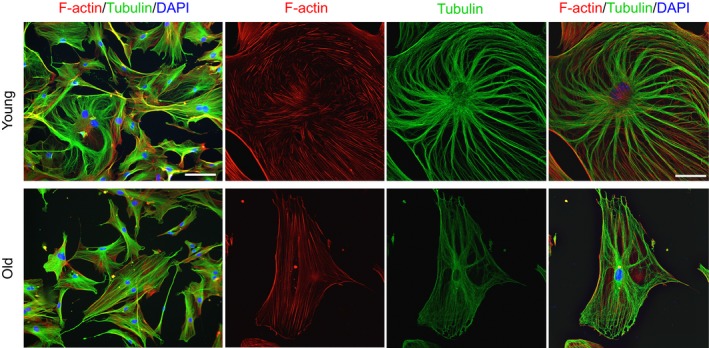
Cytoskeletal phenotype of young and old skeletal muscle fibroblasts. Fibroblasts isolated from old skeletal muscle demonstrate a contracted phenotype and increased cytoskeletal alignment as compared to those isolated from young skeletal muscle (green = tubulin; red = F‐actin; blue = DAPI). Bar = 100 μm for first column only and 50 μm for the remaining images.

Decreased physical distortion of the ECM (i.e., increased stiffness) increases cytoskeletal tension of neighboring cells, and previous reports have demonstrated that cells exposed to stiff substrates display cytoskeletal stress fibers that are preferentially aligned in the same direction (Gupta *et al*., 2015). To determine whether the increased stiffness observed in aged skeletal muscle is associated with alterations in cytoskeletal arrangement, we next characterized the morphology of fibroblasts isolated from young and old muscle using an isolation protocol based on a previously reported technique (Mathew *et al*., 2011). Regardless of age, >90% of isolated cells expressed Tcf4 (transcription factor 7‐like 2; Fig S2, Supporting information), shown to be a marker for muscle connective tissue fibroblasts (Mathew *et al*., 2011). Qualitatively, fibroblasts isolated from aged mice revealed a contracted phenotype with an increased cytoskeletal alignment (Fig 2).

Given that the cytoskeleton transmits forces to the nucleus that modulate nuclear shape and form, we next used a custom algorithm to calculate the nuclear aspect ratio (NAR) of young and old fibroblasts. The NAR is the ratio of the maximum nuclear diameter to minimum nuclear diameter. Analysis of between 475 and 500 cells per group revealed that the observed increase in cytoskeletal alignment in aged fibroblasts was accompanied by an increased nuclear elongation (NAR = 1.86 ± 0.22), when compared to young counterparts (NAR=1.67 ± 0.25; *P* = 0.04).

As predicted from tensegrity models (Ingber, 2006), altered cytoskeletal arrangement and nuclear deformation drive alterations in spatial positioning of chromatin and chromosomes to ultimately affect cellular gene expression (Thomas *et al*., 2002). To test whether age‐related alterations in fibroblast nuclear morphology were concomitant with changes in gene expression, the gene expression profile of young and aged fibroblasts was assessed using an ECM‐focused transcript array. Indeed, aged fibroblasts demonstrated a decrease expression in Col3a1 (Fig S3, Supporting information), consistent with the decreased protein staining of collagen type III in Fig S1 (Supporting information). This was concomitant with an increase in matrix metalloproteinase (MMP)‐1, which contributes to collagen 3 degradation (reviewed in Van Doren, 2015). Conversely, Col4a3 became highly expressed in aged fibroblasts (Fig S3, Supporting information). Tissue inhibitors of metalloproteinases (TIMP) 1 and 2, which promote ECM accumulation through a decreased protease activity (Brew & Nagase, 2010), were both increased in fibroblasts isolated from aged skeletal muscle (Fig S3, Supporting information). These findings suggest that aging drives matricellular gene expression changes in fibroblasts.

### The aged ECM drives a fibrogenic conversion of MuSCs at the expense of myogenicity

Next, we investigated the effect of age‐associated alterations in the skeletal muscle ECM on MuSC fate. To isolate skeletal muscle ECM, the gastrocnemius muscle obtained from young and old mice were decellularized in 1% SDS using a protocol modified from Perniconi *et al*. (2011). This decellularization protocol maintains the molecular and topological complexity characteristic of the three‐dimensional native ECM as a substrate for cell seeding. Human MuSCs were seeded onto the decellularized ECM constructs for 3 or 7 days in differentiation medium, and the differentiation of cells was quantified according to the expression of fibrogenic (ERTR7) or myogenic (desmin) markers. At the start of the experiment, MuSCs were confirmed to be 93.4% Pax7+ and 99.3% MyoD+ (Fig S4, Supporting information); 8.2% were desmin+ and 6.4% were Tcf4+ (fibroblast marker).

**Figure 3:**
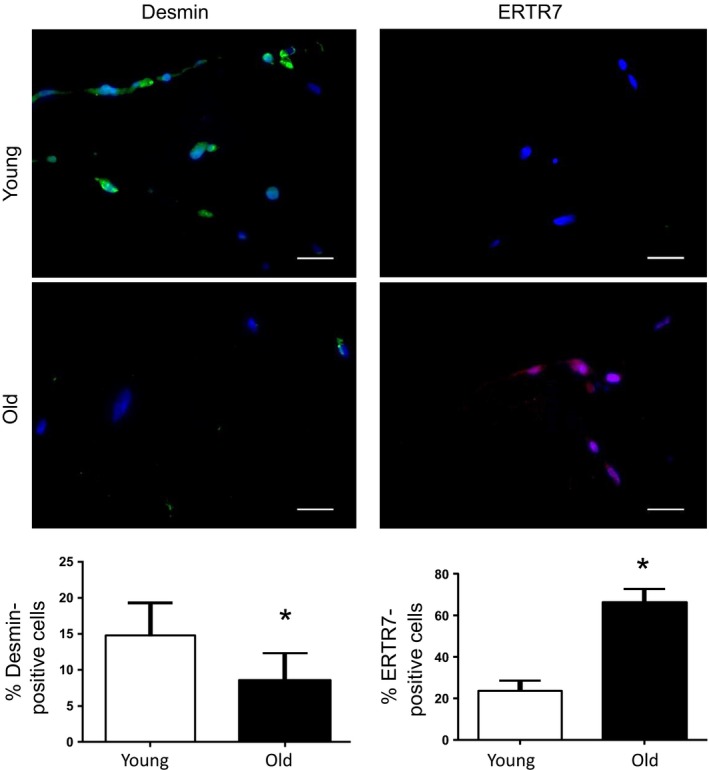
Evaluation of the direct effect of the aged ECM on MuSC fate. Immunofluorescence analysis of desmin (green) and ERTR7 (red) expression of hMuSCs seeded onto decellularized ECM for 3 days. There was a significantly greater percentage of cells stained positive for ERTR7 (young = 23.8 ± 4.8%; old = 66.3 ± 6.5%; *P < 0.001) when seeded onto aged, as compared to young, ECM constructs. Conversely, there was a significantly lower percentage of cells stained positive for desmin (young = 14.8 ± 4.5%; old = 8.5 ± 3.9%; *P = 0.049) when seeded onto aged ECM constructs, as compared to young. Bar = 100 μm.

When cells were seeded onto ECM derived from aged muscle for 3 days, we observed a significant increase in the percentage of cells expressing ERTR7, when compared to cells seeded onto young ECM substrates (Fig 3). Conversely, we observed a significant decrease in the number of desmin‐positive cells when seeded onto aged ECM constructs, as compared to cells seeded onto young ECM constructs (Fig 3). Interestingly, after 3 days in culture, only 38.7% of the cells analyzed expressed either ERTR7 or desmin markers when seeded onto young ECM constructs, whereas 74.8% of the cells expressed markers of fibrogenic differentiation when seeded onto aged constructs, with an average of only 5.2% of the cells coexpressing ERTR7 and desmin. Gilbert *et al*. (2010) demonstrated that MuSCs seeded onto soft substrates display increased stemness, as evidenced by an increase in the expression of Pax7 (Gilbert *et al*., 2010). It is possible that cells seeded onto young ECM constructs persist in a more stemlike state, compared to those seeded onto aged ECM counterparts that may more rapidly differentiate.

When we allowed cells to differentiate for 7 days on ECM constructs, the percentage of desmin+ cells observed on young ECM constructs increased to 30.3 ± 6.0%, whereas cells seeded onto aged constructs were 15.4 ± 4.7% desmin‐positive (*P* = 0.02). After 7 days, >70% of cells seeded onto both young and aged ECM displayed markers of either a myogenic or fibrogenic lineage. The remaining cells were negative for both desmin and ERTR7, suggesting these cells had either differentiated toward another lineage (e.g., adipogenic) or were undifferentiated.

We cannot rule out the possibility that differences in fate of MuSCs seeded onto old vs. young ECM may be due to changes in the biochemical composition of the ECM that occur with age. Therefore, to evaluate the direct effect of age‐related ECM biochemical alterations on muscle stem cell lineage progression, the ECM of young and old animals was once again isolated using a decellularization protocol. This time, however, the ECM was pulverized and solubilized by urea extraction and the solubilized preparations from young or old animals were subsequently used to coat chamber slides. When MuSCs were seeded onto an aged matrix substrate, we once again observed a dramatic decrease in the percentage of myogenic markers and a concomitant increase in Tcf4 expression, as compared to cells that were seeded onto an ECM substrate derived from young skeletal muscle (Fig S5, Supporting information). These findings suggest that ECM compositional changes associated with aging of the ECM play a role in promoting MuSC fibrogenesis.

**Figure 4:**
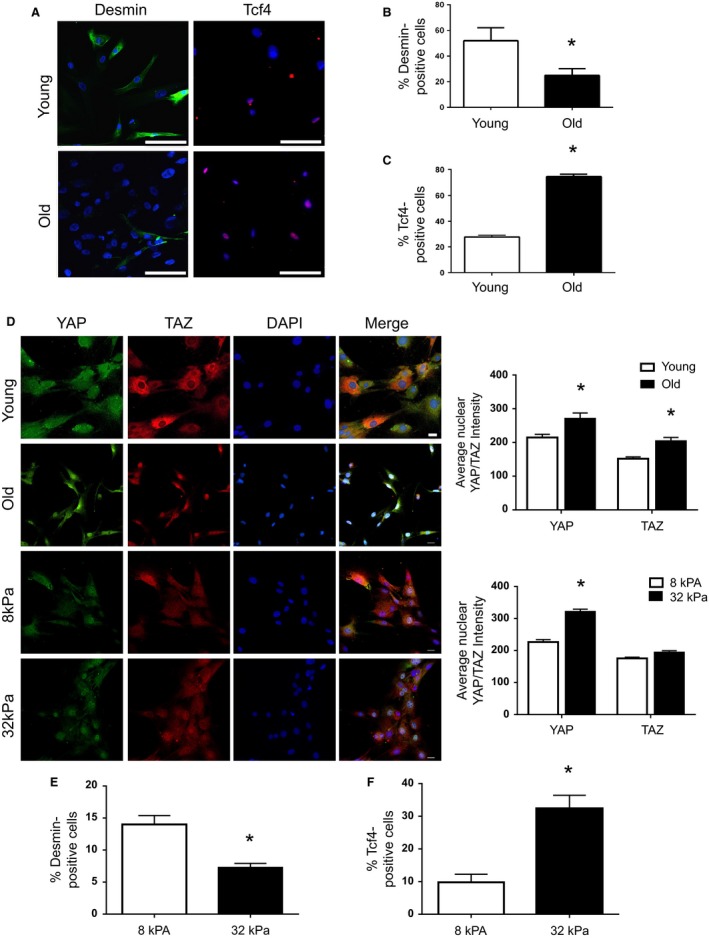
The effect of age and substrate stiffness on YAP/TAZ signaling in fibroblasts and its influence on MuSC fate. MuSCs cultured on matrices derived from old fibroblasts demonstrated a significant decrease in the percentage of desmin‐positive cells (young = 26.8 ± 1.5%; old = 11.9 ± 1.9%; *P < 0.0001) and a significant increase in the percentage of Tcf4‐positive cells (young = 27.8 ± 1.4%; old = 74.5 ± 1.2%; *P < 0.0001), relative to MuSCs cultured on matrices derived from young fibroblasts (A‐C). Representative immunofluorescence images of YAP/TAZ expression from old and young fibroblasts, and from young fibroblasts cultured on soft (8 kPA) and stiff (32 kPA) silicone gel substrates. Compared to young fibroblasts, old fibroblasts demonstrate a significant increase in the nuclear translocation of both YAP (young = 214.7 ± 9.5; old = 271.1 ± 16.5; *P = 0.0003 and TAZ (young = 152.0 ± 5.3; old = 204.5 ± 10.3; *P = 0.0008). Similarly, young fibroblasts cultured on stiff substrates exhibited a significant increase in YAP/TAZ nuclear translocation, relative to those cultured on soft substrates. When these preconditioned fibroblasts were allowed to elaborate a matrix for 2 days, cells previously cultured on the stiff substrate demonstrated a significant decrease in the percentage of desmin‐positive cells (soft surface = 14.0 ± 1.4; stiff surface 7.3 ± 0.6; *P* = 0.0004), and a significant increase in the percentage of Tcf4‐positive cells (soft surface = 10.78 ± 1.0; stiff surface 32.3 ± 1.4; *P* = 0.0008), consistent with that observed in fibroblasts cultured on young as compared to old ECM (E, F). Bar = 100 μm.

Given the aforementioned ECM‐related gene expression alterations observed in aged fibroblasts, we tested whether matrices elaborated by aged fibroblasts are inhibitory for MuSC myogenicity. Young and aged fibroblasts were allowed to elaborate a matrix in culture for 2 days, after which time the fibroblasts were lysed. The decellularized substrate from each group was subsequently seeded with MuSCs and subjected to differentiation‐promoting conditions for 3 days. There was both a decrease in desmin expression and an increase in the number of Tcf4‐positive cells when cells were seeded onto aged fibroblast‐derived matrix, as compared to cells seeded onto a young fibroblast‐derived matrix. (Fig. 4A–C).

We postulated that the increased stiffness of the aged skeletal muscle ECM drives pathogenic matrix secretion by fibroblasts via mechanotransductive pathways. To first investigate this hypothesis, old and young fibroblasts were cultured on collagen‐coated plates for 3–4 days and then fixed for evaluation of YAP/TAZ expression. YAP/TAZ are transcriptional regulators that relay mechanical signals imposed by the matrix and act as mediators of mechanotransductive signaling (Dupont *et al*., 2011; Aragona *et al*., 2013). Indeed, we find that aged fibroblasts display an increased nuclear translocation of YAP/TAZ (Fig 4D). The increased nuclear YAP/TAZ of isolated aged fibroblasts is consistent with our findings of increased muscle stiffness with aging (Fig 1) and is in accordance with previous reports demonstrating an increased nuclear YAP/TAZ expression in response to a pathologically stiffened ECM (Liu *et al*., 2015).

**Figure 5:**
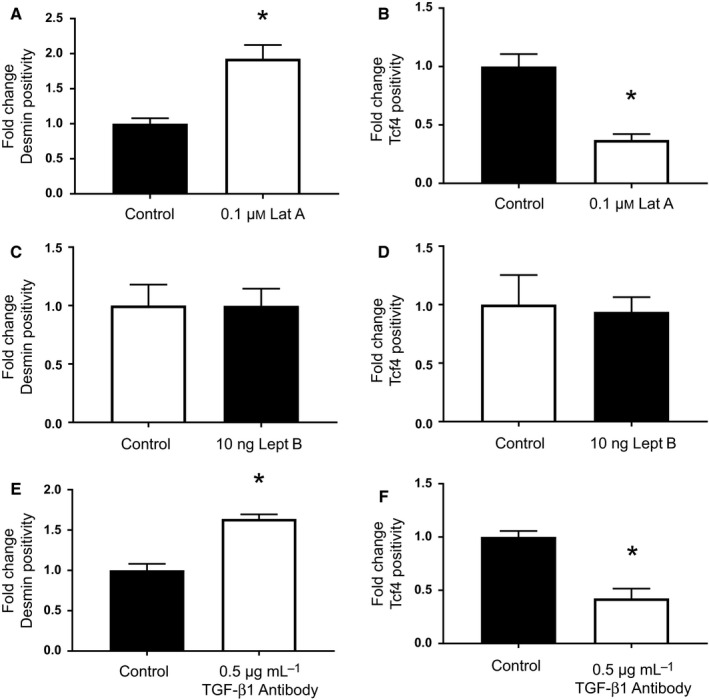
The effect of fibroblast YAP/TAZ and TGF‐B1 modulation on MuSC fate. Pharmacologic inhibition of F‐actin polymerization in young myofibroblasts with latrunculin A resulted in a significant increase in the percentage of desmin‐positive cells (*P < 0.0001), and a significant decrease in the percentage of Tcf4‐positive cells (*P < 0.0001), relative to untreated cells (A, B). No significant difference in the percentage of desmin‐ or Tcf4‐positive cells was observed following treatment with leptomycin B (C, D). As with latrunculin A treatment, pharmacologic inhibition of TGF‐β1 resulted in a significant increase in the percentage of desmin‐positive cells (*P < 0.0001), and a significant decrease in the percentage of Tcf4‐positive cells (*P < 0.0001), relative to untreated cells (E, F).

These findings then prompted us to ask whether manipulation of the microenvironment onto which young fibroblasts were cultured could induce elaboration of an ‘aged’ matrix. To test this, young fibroblasts were preconditioned on a soft (elastic modulus (λ) = 8 KPa) or stiff (λ = 32 KPa) silicone gel substrate for 3 days. As expected, young fibroblasts seeded on a stiff substrate displayed a significantly increased nuclear translocation of YAP/TAZ, as compared to young counterparts seeded onto a soft substrate (Fig 4D). Remarkably, young fibroblasts exposed to a stiff substrate *in vitro* display a matricellular protein secretion that favors a fibrogenic conversion of MuSCs and an impaired myogenicity, similar to the matrix elaborated by aged fibroblast counterparts (Figs 5E, F and S6, Supporting information).

These data suggest that matricellular protein expression by fibroblasts is responsive to mechanical signals from the surrounding microenvironment via YAP/TAZ signaling. Given previous studies demonstrating that cytoskeletal tension is required for nuclear translocation of YAP/TAZ (Dupont *et al*., 2011; Aragona *et al*., 2013), we next evaluated whether pharmacologic inhibition of F‐actin polymerization was sufficient to at least partially reverse the aged fibroblast phenotype. Indeed, treatment of aged fibroblasts with the F‐actin inhibitor, latrunculin A, decreased nuclear YAP/TAZ expression (Fig S7A, Supporting information) and promoted secretion of a matrix by aged fibroblasts that favored MuSC myogenicity and inhibited fibrogenesis, when compared to untreated counterparts (Fig 5A,B). Consistent with previous reports (Dupont *et al*., 2011), we found that blockade of nuclear export in young fibroblasts by leptomycin B at a dose of 10 ng significantly increased nuclear YAP/TAZ expression (Fig S7B, Supporting information). However, treatment of young myofibroblasts with leptomycin B at the dosage tested was not sufficient to drive a pro‐fibrogenic matrix (Fig 5C,D).

Finally, because recent studies have highlighted the convergence of YAP/TAZ proteins and transforming growth factor‐beta (TGF‐β1) signaling pathway (reviewed in Piersma *et al*., 2015), we evaluated whether pharmacologic inhibition of TGF‐β1, a master regulator of fibrogenesis, could reverse the effect of age on fibroblast matricellular protein expression. Indeed, MuSCs seeded onto a matrix derived from aged fibroblasts treated with a TGF‐β1 neutralizing antibody displayed an increase in myogenicity and a decreased expression of Tcf4, when compared to untreated counterparts (Fig 5E,F).

Taken together, the results of this study demonstrate that aging of the ECM drives nuclear translocation of YAP/TAZ and a pathogenic matricellular protein expression by fibroblasts. This aged matricellular profile, in turn, disrupts MuSC lineage specification. These findings suggest a possible mechanism for the previously reported myogenic‐to‐fibrogenic conversion of aged MuSCs and increased fibrosis formation of aged muscle following an acute injury.

## Discussion

The skeletal muscle microenvironment is increasingly recognized as playing a critical role in the determination of skeletal muscle regenerative capacity. While a series of elegant studies have implicated disease and age‐related alterations in the MuSC niche as contributing to declines in skeletal muscle regenerative potential through a myogenic‐to‐fibrogenic conversion of MuSCs (Conboy *et al*., 2005; Brack *et al*., 2007; Biressi *et al*., 2014; Zordan *et al*., 2014; Pessina *et al*., 2015), studies have largely focused on circulating factors as primary culprits. Although the ECM constitutes an undeniably important component of the MuSC niche, our understanding of age‐related ECM alterations and whether these contribute to the decreased MuSC regenerative potential with aging remains poorly understood. The findings from the current study demonstrate that aging drives alterations in ECM biophysical properties, alterations that are likely to directly affect the gene expression profiles of resident fibroblasts responsible for the secretion of matricellular components through YAP/TAZ signaling. The resulting matricellular profiles may contribute to the aged‐related changes in ECM that promote MuSC fibrogenic conversion.

Analysis of collagen fibril structure and architecture in the skeletal muscle ECM of young and old mice revealed that aged ECM displays a significant decrease in collagen fiber tortuosity, when compared to young muscle counterparts. A decreased collagen fibril tortuosity with increasing age and, therefore, a decreased compliance in response to tensile loading should render the muscle stiffer along the axis of the fibril alignment (i.e., the circumferential direction). While not previously investigated in skeletal muscle, similar age‐related alterations in collagen fibril topography, as determined by scanning and transmission electron microscopy, in aging skin have been reported (Imayama & Braverman, 1989). These studies revealed that young skin displays an ordered arrangement of elastic fibers and a concurrent tortuous collagen fibril alignment (Imayama & Braverman, 1989). However, with increasing age, collagen bundles become more taut, causing decreased connective tissue compliance. Likewise, our finding of decreased collagen fiber tortuosity in aged muscle was consistent with the increased muscle stiffness observed in the circumferential direction during biaxial mechanical testing. Previous studies have also demonstrated increased skeletal muscle stiffness with increasing age in both rodents (Alnaqeeb *et al*., 1984; Gosselin *et al*., 1994; Wood *et al*., 2014) and humans (Blanpied & Smidt, 1993). However, the bulk of previously published studies determined muscle mechanical properties using uniaxial systems. While the predominant line of action of the gastrocnemius is caudal–cephalic, uniaxial mechanical testing does not allow for the assessment of out‐of‐plane properties of biologic tissue, nor does it allow for the detection of potential changes in tissue mechanics dictated by alterations in the three‐dimensional microarchitecture. The biaxial results in this study provide additional directional information on muscle structural alterations due to age and corroborate our SHG findings.

### Implications for age‐related alterations in ECM mechanical properties on fibroblast and MuSC function

An increased stiffness of the whole muscle is expected to hamper the total myofiber displacement during contraction and relaxation. As such, mechanical properties at the macroscopic scale may influence structural rearrangements of the interconnected cytoskeletons. The increased stiffness of the aged ECM relative to young counterparts, as observed with biaxial testing (Fig 1), is concomitant with the cytoskeletal remodeling and nuclear elongation in the aged fibroblasts (Fig 2).

We also observed alterations in key matrix proteins of aged fibroblasts, which may help explain changes in the mechanical properties of skeletal muscle ECM with aging. Overall, there was decreased expression of several collagen genes, including Col3a1 and Col6a1. The observed decrease in Col3a1 expression (a highly compliant collagen subtype) is consistent with both our histological findings and the increased stiffness observed during biaxial mechanical testing. The decreased expression of Col6a1 has potentially important implications for MuSC function as a lack of collagen type VI has been demonstrated to impair muscle regeneration and reduce satellite cell self‐renewal capacity after injury in mice (Urciuolo *et al*., 2013).

Nearly three decades ago, Ingber and colleagues suggested that cell fate may be controlled via mechanical mechanisms within the tissue (Ingber *et al*., 1981, 1985). More recently, using two‐dimensional culture systems in which substrate stiffness was engineered to mimic tissues with varying matrix rigidities, including brain, muscle, and bone, the fate of mesenchymal stem cells was powerfully modulated according to elastic characteristics of substrate upon which the cells were maintained (Engler *et al*., 2006). Planar culture conditions that were engineered to mimic the elastic modulus typical of adult, healthy skeletal muscle promoted MuSC maintenance of ‘stemness’ and myogenic potential (Gilbert *et al*., 2010). There is also *in vivo* evidence to suggest that ECM mechanical properties may affect stem cell fate determination. Dystrophic skeletal muscle, which is also characterized by a significant decrease in tissue compliance (average elastic modulus of 18 kPa, range from 5 to 35 kPa (Engler *et al*., 2004)), also displays MuSC dysfunction and an impaired muscle regenerative capacity (Biressi *et al*., 2014; Pessina *et al*., 2015). However, modulation of the dystrophic skeletal muscle microenvironment through the transplantation of cells engineered to both promote angiogenesis and decrease fibrosis results in a significant improvement in muscle regeneration and function (Gargioli *et al*., 2008). These findings further confirm a close relationship between the physical microenvironment and stem cell behavior, even *in vivo*.

**Figure 6:**
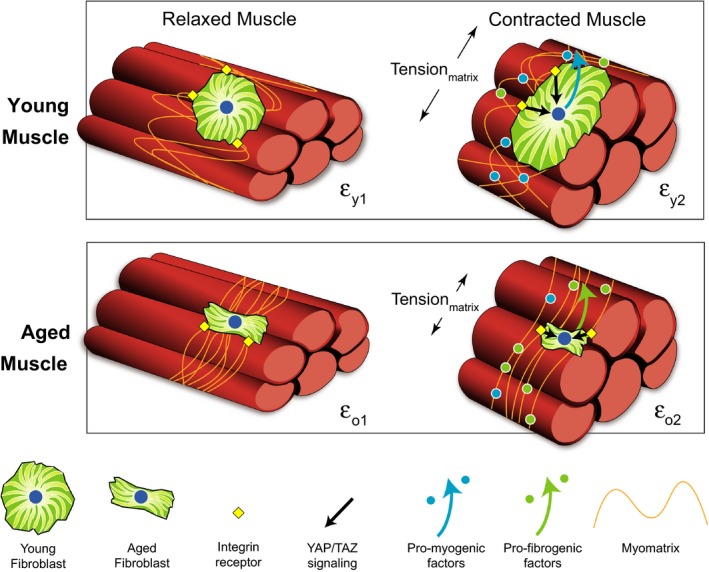
Proposed hypothesis schematic. Tortuous collagen fibrils in the young myomatrix are compliant and experience deformation (ε_y_) with loading during muscle contraction. The compliance of the young ECM triggers mechanotransductive signaling in fibroblasts via integrins to promote the secretion of biochemical matrix factors that are more favorable for muscle stem cell myogenesis. Conversely, in aging, collagen fibrils become more taut and display a decreased deformation in response to contractile activity (ε_o_; ε_y_ > ε_o_), thereby rendering the muscle more stiff. The resulting mechanotransductive cascade in fibroblasts increases nuclear translocation of YAP/TAZ and triggers the expression of matrix‐associated biochemical factors that promote muscle stem cell fibrogenesis.

While previous investigations have probed the effect of aging on ECM characteristics, there is little information as to how age‐related changes in the ECM and associated molecules, including signaling factors, may affect skeletal muscle regenerative potential and, more specifically, MuSC function. Indeed, our current findings reveal a potent effect of the aged ECM on MuSC fate. Exposure of human‐derived MuSCs to an aged myomatrix inhibited myogenicity and promoted a fibrogenic conversion of cells, when compared to cells seeded onto similarly prepared decellularized matrix constructs derived from young skeletal muscle. Although age‐related alterations in ECM biophysical properties may play a role in dictating stem cell behavior, our findings suggest that the effect of the aged ECM on MuSC fate is also attributed to alterations in the secretion of soluble factors by aged fibroblasts, shown in our studies to inhibit MuSC myogenicity. Our working model proposes that an age‐related increase in muscle rigidity drives activation of the downstream mechanotransductive pathway, YAP/TAZ, to modulate fibroblast expression of matrix‐associated proteins, proteins which subsequently inhibit MuSC myogenicity in favor of a fibrogenesis (Fig 6). These findings lend insight into the well‐established decline in skeletal muscle regeneration accompanying the aging process and implicate the skeletal muscle ECM as a novel therapeutic target in the development of strategies to enhance skeletal muscle regenerative capacity in an aged population.

## Experimental procedures

All animal studies were conducted in compliance with the US Department of Health and Human Services Guide for the Care and Use of Laboratory animals and approved by the University of Pittsburgh IACUC (IACUC # 15035334).

### Multiphoton imaging and characterization of ECM topography and architecture

The gastrocnemius muscle was excised and placed in 2% paraformaldehyde for 2 h, and then in scaleview solution for at least 2 weeks. An Olympus multiphoton microscope (Model FV10; ASW Software, Olympus, Center Valley, Pennsylvania, USA) was used to observe elastin and collagen fibers, as previously described (D'Amore *et al*., 2010; Koch *et al*., 2014). From images, the fiber orientation index and tortuosity were calculated using custom MATLAB‐automated image‐based analysis tools (D'Amore *et al*., 2010; Koch *et al*., 2014).

### Muscle decellularization and histological analysis

Using a protocol adapted from Perniconi *et al*. (2011), gastrocnemius muscles from young and old mice were decellularized for 48 h in 1% SDS in diH_2_0. After 48 h, serial rinses in PBS and DiH20 were performed for 30 min each for a total of 2 h. The decellularized matrix was then (i) fixed in 10% neutral buffered formalin and embedded in paraffin or (ii) placed in differentiation medium (DMEM containing 10% FBS and 1% P/S) for use in cell seeding experiments. Hematoxylin/eosin and DAPI were used to confirm the absence of nuclear material. Herovici and Verhoeff–van Gieson stains were used to observe the abundance and distribution of collagen III and elastin, respectively. The abundance of each component was quantified using NIS‐Elements.

### Biaxial testing

Biaxial mechanical testing of intact skeletal muscle was performed as previously described (Hashizume *et al*., 2010). Briefly, gastrocnemius muscles were harvested from young (*n* = 7) and old (*n* = 7) animals and placed in chilled ringers solution for 1 h prior to testing. The Achilles tendon was removed, and the lateral gastrocnemius was dissected and isolated for biaxial testing. According to a well‐established method (Sacks & Sun, 2003), square samples (~7 mm × 7 mm) were affixed to 250 *g* load cells (Model 31, Honeywell, Columbus, OH, USA) with two loops of suture attached to each side with four hooks. Tissue deformation was measured by processing and mapping real time the coordinates of a four‐marker array (1 mm diameter) positioned in the central 4 × 4 mm region of the specimen. The resulting deformation gradient tensor F was computed, from which the axial stretches *Fii* were determined. Equi‐stress protocol was then performed with the samples submersed in PBS solution at room temperature utilizing a tare load of 0.5 *g* and a cycle time (loading and unloading) of 30 s. Constructs were preconditioned and tested up to the determined maximum stress of 30 kPa, a critical load that was determined to be the maximum stress value that native muscle tissue is able to withstand without incurring permanent damage. Therefore, the strain range investigated (0–12%) covered both physiological and overphysiological levels of tissue deformation. All data were referenced to the postpreconditioned free‐float state (D'Amore *et al*., 2014, 2016).

### Isolation of fibroblasts

Fibroblasts were isolated from the hindlimb muscles of young (3–4 months old) and old (22–24 months old; NIA Rodent Colony, Bethesda, Maryland, USA) C57Bl/6 mice by digestion with 1000 U mL^−1^ collagenase XI for 60 min, followed by digestion with dispase for 45 min and then digestion with 0.1% trypsin for 30 min at 37 °C. Cells were pelleted and resuspended in Dulbecco's modified Eagle's medium (DMEM) containing 10% fetal bovine serum (FBS) and 1% of penicillin–streptomycin (P/S). The homogenate was filtered through a 70‐μm strainer and plated onto collagen‐coated flasks for 24 h, after which time the unattached cells were removed and the flasks were filled with fresh medium. Cells were grown for an additional 48–72 h and used for experiments.

Cells were plated on chamber slides, washed with PBS, fixed in 2% paraformaldehyde (PFA) for 20 min, permeabilized with 0.03% Triton X‐100 for 20 min, and washed twice in phosphate‐buffered saline (PBS). Nonspecific binding was blocked for 1 h using 3% goat serum in PBS. Afterward, cells were incubated with mouse anti‐β‐tubulin antibody (1:500; Rockland, Limerick, PA, USA) overnight at room temperature. Following three PBS washes, cells were double‐stained with AlexaFluor 488 goat anti‐mouse secondary antibody (1:1000; Life Technologies, Grand Island NY, USA) and AlexaFluor 594 Phalloidin (1:1000; Life Technologies) for 45 min. Samples were washed again three times with PBS and mounted with Fluoromount G containing DAPI (4′‐6‐diamidino‐2‐phenylindole). Cells were imaged using an inverted confocal microscope (Nikon A1; Nikon, Melville, New York, USA) controlled by NIS‐Elements software.

### Quantification of cytoskeletal alignment and nuclear aspect ratio

The nuclear aspect ratio (NAR) was calculated for all nuclei within one image as previously described (Stella *et al*., 2008; D'Amore *et al*., 2014).

### ECM array of tissue and fibroblasts

Total RNA was isolated from whole‐muscle tissue or cultured fibroblasts using TRIzol (ThermoFisher, Pittsburgh, PA, USA) following the manufacturer's recommendations. For qPCR array, cDNA was synthesized using the RT^2^ First Strand Kit (Qiagen, Germantown, MD, USA) with the recommended 1 μg of total RNA. The cDNA was loaded and amplified on the RT^2^ Profiler PCR Array for mouse ECM and adhesion molecules (Qiagen, Germantown, MD, USA). The data underwent quality control and analysis using the associated online software. Transcript values were normalized to Gusb (beta‐glucuronidase), a housekeeping gene showing no change between groups, and reported as the fold changed in transcript expression in the old muscle relative to young.

### Cell seeding onto young and old decellularized ECM

Human muscle satellite cells (hMuSCs; ScienCell Inc., Carlsbad, CA, USA) were cultured in growth media (DMEM containing 10% FBS, 10% HS, 0.5% chick embryo extract (CEE; MP Biomedicals, Santa Ana, CA, USA), and 1% P/S). On the day of cell seeding, hMuSCs were trypsinized and resuspended in low‐serum medium (DMEM containing 10% FBS and 1% P/S) at a concentration of 3 × 10^6^ per mL, and seeded onto the muscle matrix derived from decellularized gastrocnemius muscles. After 3 or 7 days, hMuSC‐ECM cultures were fixed in 2% PFA for 20 min and transferred to 30% sucrose solution for 24 h. The fixed tissues were then snap‐frozen in liquid nitrogen‐cooled 2‐methylbutane. Samples were then cryosectioned (7 μm) and mounted on glass slides. Sections were incubated overnight with either rat anti‐Er‐Tr7 antibody (1:500; Hycult, Plymouth Meeting, PA, USA) or rabbit anti‐desmin antibody (1:1000; Abcam, Cambridge, MA, USA), followed by a 45‐min incubation with AlexaFluor 594 goat anti‐rat or AlexaFluor 488 goat anti‐rabbit secondary antibodies (1:1000; Life Technologies).

### Preparation of solubilized ECM extract and cell culture

Appendicular muscles from 10 young and 10 old mice were harvested and stored at −20 °C in a protease inhibitor solution composed of 1X PBS (Gibco, Grand Island, NY, USA) supplemented with 5 mm ethylenediaminetetraacetic acid (EDTA; Sigma‐Aldrich, St. Louis, MO, USA) and 0.5 mm phenylmethylsulfonyl fluoride (PMSF; Sigma‐Aldrich). Muscle from young and old animals was equally divided and prepared in duplicate (*n* = 2 per age), and a water‐soluble fraction of muscle ECM was prepared as previously described (Yang *et al*., 2013). Solubilized ECM extracts were used to coat the bottom of chamber slides at 50 μL per well. 2 × 10^4^ human MuSCs/chamber were plated and incubated for 3 days. Cells were then stained with rabbit anti‐Tcf4 antibody (1:500; Cell Signaling, Danvers, MA, USA) or desmin, as described above.

### Cell seeding onto matrices elaborated from young and old fibroblasts

Fibroblasts isolated from young or aged skeletal muscle were seeded onto chamber slides (10 000 cells per well) and cultured in growth medium (as above) for 4 days. Fibroblasts were then lysed by exposure to distilled water for 1 h, followed by three washes with PBS. DAPI staining confirmed the absence of living cells. Human MuSCs were then seeded onto the elaborated matrices and allowed to expand for 3 days in low‐serum media, followed by staining with desmin or Tcf4, as described above.

### Fibroblast characterization following culture onto soft vs. stiff substrates

Fibroblasts isolated from young skeletal muscle were seeded onto soft (λ = 8 KPa) or stiff (λ = 32 KPa) silicone gel 6‐well plates (Advanced Biomatrix, San Diego, CA, USA) at a density of 20 000 cells per well. Cells were cultured in growth medium to 70–80% confluence (2–3 days). Cells were then trypsinized, seeded onto collagen‐coated 8‐well chamber slides, and allowed to elaborate a matrix for 4 days, after which time cells were fixed and immunocytochemistry was performed to quantify expression of collagens III, IV, and VI.

In a second series of experiments, young muscle‐derived fibroblasts were again seeded onto soft or stiff silicone gel 6‐well plates as described above. After 4 days, cells were lysed and 2 × 10^4^ human MuSCs were seeded onto the elaborated matrices and MuSC expression of Tcf4 and desmin was quantified as above.

### Quantification and modulation of nuclear YAP/TAZ

Young muscle‐derived fibroblasts were seeded onto soft or stiff silicone gel 6‐well plates and cultured as described above. Cells were then trypsinized and seeded (10 000 cells per well) onto collagen I‐coated 8‐well chamber slides (Lab Tek II; ThermoFisher Scientific, Canoga Park, CA, USA). After 3–4 days, cells were fixed with 2% PFA and immunocytochemistry was performed to quantify expression of nuclear YAP (Sigma‐Aldrich sc101199) and nuclear TAZ (Sigma‐Aldrich T4077) using an automated custom‐designed macro to quantify YAP and TAZ only in the areas colocalized with DAPI (NIS‐Elements). Similar YAP/TAZ quantification was performed for young and aged fibroblasts.

Aged fibroblasts were seeded onto chamber slides as above for 2 days, after which time cells were treated with 0.1 μm of latrunculin A. After 1 h, cells were washed with media and incubated for an additional 2 days in proliferation medium. Fibroblasts were lysed and the conditioned medium was seeded with hMuSCs, as above. To promote nuclear YAP/TAZ accumulation in young fibroblasts, cells were plated as above and subsequently treated with 40 ng of leptomycin B for 30 min. To evaluate the effect of TGF‐β1 inhibition of the fibroblast matrisome, aged fibroblasts were cultured for 2 days and then treated with 0.5 μg mL^−1^ of TGF‐β1 neutralizing antibody and incubated for an additional 2 days.

### Statistics

All statistical analysis was performed using spss software (Armonk, New York, USA) (*P* < 0.05). Independent‐samples *t*‐tests were used to evaluate between group differences for all variables of interest, except for peak ECM stiffness in the longitudinal and circumferential directions and the anisotropy ratio, which were evaluated using a one‐way ANOVA. Data are presented as mean ± SEM, unless otherwise noted.

## Funding

This work was supported by the NIH NIA Grant K01AG039477 (FA), NIEHS Grant F32ES022134 (KB), NIEHS Grant R01ES023696 (FA and AB), NIEHS Grant R01ES025529 (FA and AB), and the University of Pittsburgh Medical Center Rehabilitation Institute.

## Author contributions

Drs. Stearns‐Reider and Ambrosio provided concept/idea/research design and writing. Drs. Stearns‐Reider, Beezhold, D'Amore, Rothrauff, Cavalli, Zhang, and Ms. Shinde provided data collection. Drs. Stearns‐Reider, D'Amore, and Ambrosio provided data analysis and writing. Dr. Ambrosio provided project management. Drs. Wagner, Vorp, Barchowsky, Rando, and Tuan provided consultation with data analysis and interpretation, including review of the manuscript before submission.

## Conflict of interest

None declared.

## Supporting information


**Fig. S1** The histomorphometric analysis of collagen type III and elastin in young and old skeletal muscle.
**Fig. S2** DAPI and Tcf4 staining of fibroblasts isolated from the skeletal muscle of young and old mice.
**Fig. S3** The microarray gene expression profiling in young and old fibroblasts.
**Fig. S4** The expression of Pax7 & MyoD in the human muscle stem cells utilized in cell seeding experiments.
**Fig. S5** The resulting expression of desmin (A) and Tcf4 (B) from MuSCs seeded onto young and old decellularized and solubilized matrices.
**Fig. S6** The analysis of collagen composition between ECM deposited by young and old fibroblasts.
**Fig. S7** The dose response of latrunculin A (A) and leptomycin B (B).
