# Supplementary material for: Aging of the skeletal muscle extracellular matrix drives a stem cell fibrogenic conversion
Source: Aging Cell. 2017 Mar 30;16(3):518–28. doi: 10.1111/acel.12578 (PMC5418187; doi:10.1111/acel.12578)

**Supplemental Figure 1: Histomorphometric analysis of collagen type III and elastin in young and old skeletal muscle.**

(A) Images of the Herovici (collagen III) and Verhoeff-van Gieson (elastin) stains from young (n=4) and old (n=4) ECM. Collagen type III (B) and elastin (C) fiber area was significantly lower in old skeletal muscle ECM, as compared to young (collagen type III: young= (% area/high power field) 37.3% ± 1.7%; old= 26.1 ± 3.3%; *p=0.02; elastin: young= 33.1% ± 2.8%; old= 26.1 ± 1.9%; *p=0.04). Bar=50μm.

**
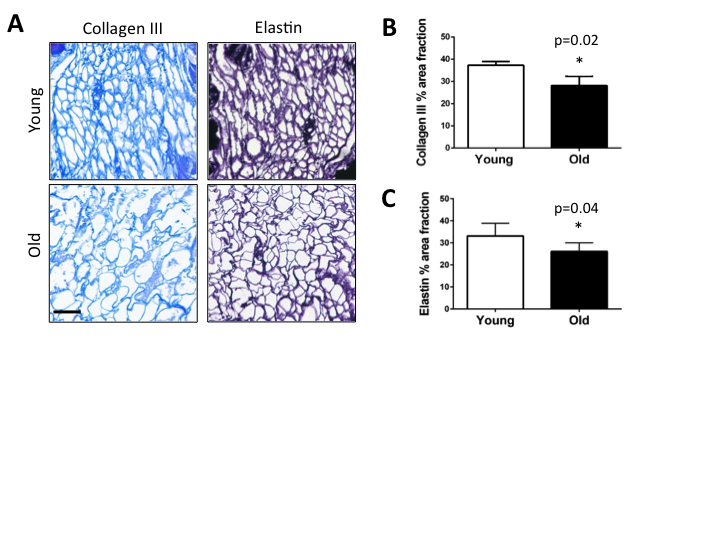
**

**Supplemental Figure 2: Myofibroblast isolation from the skeletal muscle of young and old mice.**

To confirm the presence of myofibroblasts for cell seeding experiments, cells were isolated from the hindlimb musculature of both young and old mice and cultured, as described in the Methods. Greater than 90% of cells isolated from each group expressed Tcf4.

***
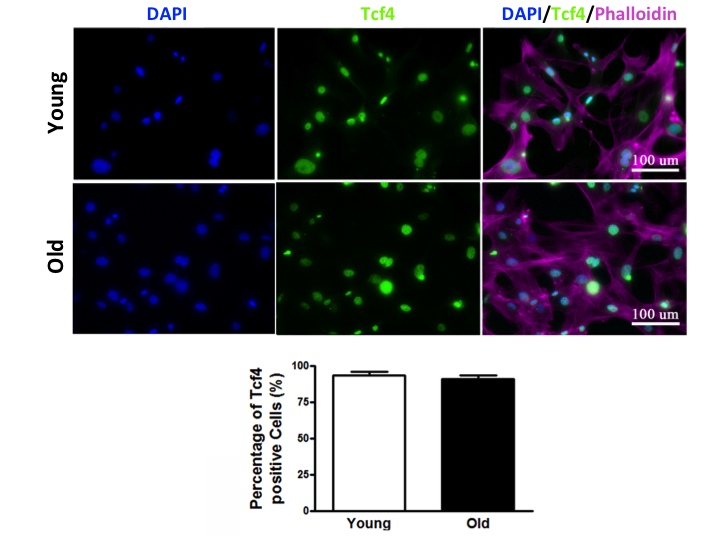
***

**Supplemental Figure 3: Microarray gene expression profiling in young and old myofibroblasts.**

The fold increase in transcript expression in cells isolated from old mice, compared to those from young mice, is shown for specific adhesion molecules, receptors, structural proteins, proteases, and other ECM components.

***
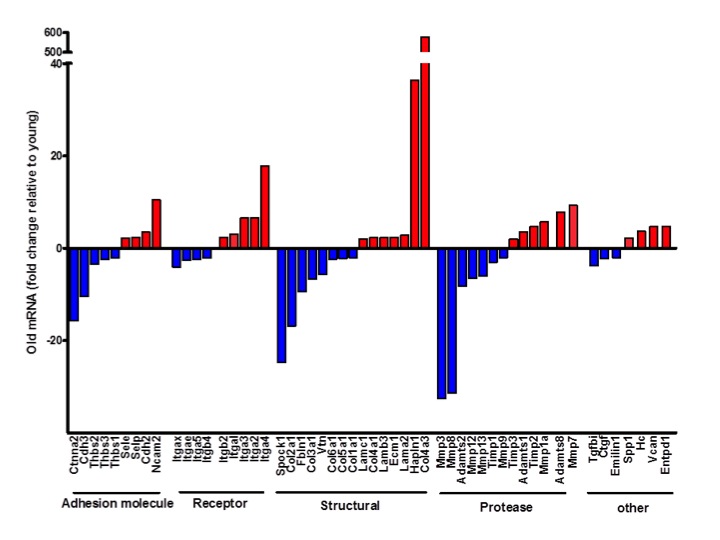
***

**Supplemental Figure 4: Characterization of Human MuSC’s utilized in cell seeding experiments.**

Images from Immunofluorescence analysis of Human MuSC’s utilized for cell seeding experiments revealed that cells were 93.4% Pax7+ (A) and 99.3% MyoD+ (B).

**
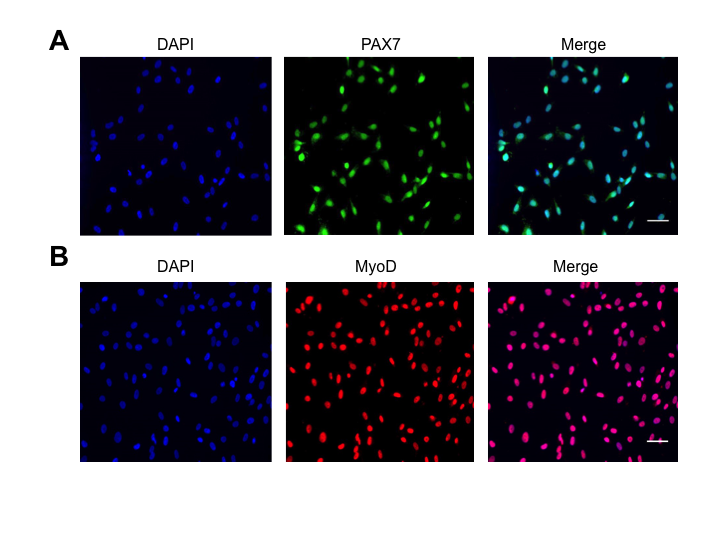
**

**Supplemental Figure 5: Seeding onto solubilized extracts of young and old skeletal muscle ECM differentially affects MuSC phenotype.**

Representative immunofluroescence images for desmin (A) and Tcf4 (B) expression of cells seeded onto young and old decellularized and solubilized matrices. MuSCs cultured onto solubilized muscle ECM from old mice showed a significant decrease in the percentage of cells expressing the myogenic marker, desmin (young= 57.0% ± 2.9%; old= 43.7% ± 1.1%; p=0.0056)(C), and a significant increase in the percentage of cells expressing the fibrogenic marker, Tcf4 (young= 49.5% ± 7.3%; old= 78.0% ± 2.3%; p=0.0094) (D). Bar=100μm.


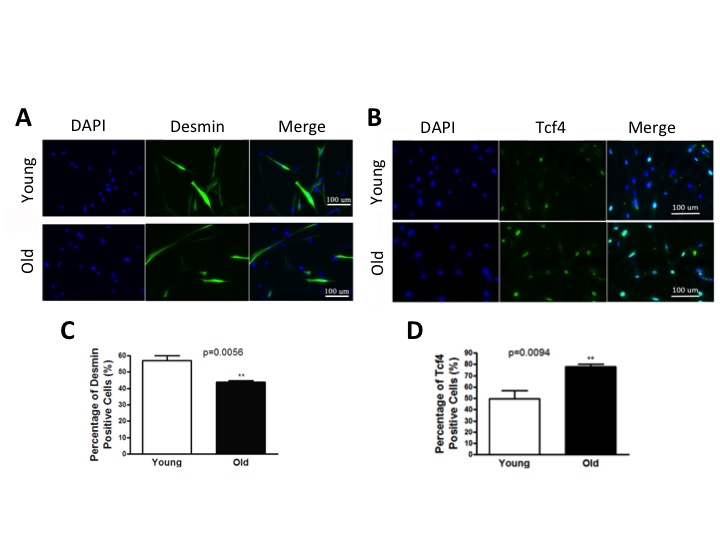


**Supplemental Figure 6: Analysis of collagen composition between ECM deposited by young and old myofibroblasts.**

Confocal imaging analysis was performed to quantify collagen intensity per nucleus. Images expressing collagen type III (red), IV (green), and VI (red) are displayed above. Significantly lower expression of collagen III and VI were observed in ECM established by old MF (p<0.0001), while ECM from young MF had higher collagen III and VI expression and significantly lower expression of Collagen IV as compared to old (p< 0.0001). We observed a similar trend when young myofibroblasts were pre-conditioned on a soft (Elastic modulus (λ) = 8 KPa) or stiff (λ = 32 KPa) silicone gel substrate for three days and then allowed to form matrix on chamber slides. t**
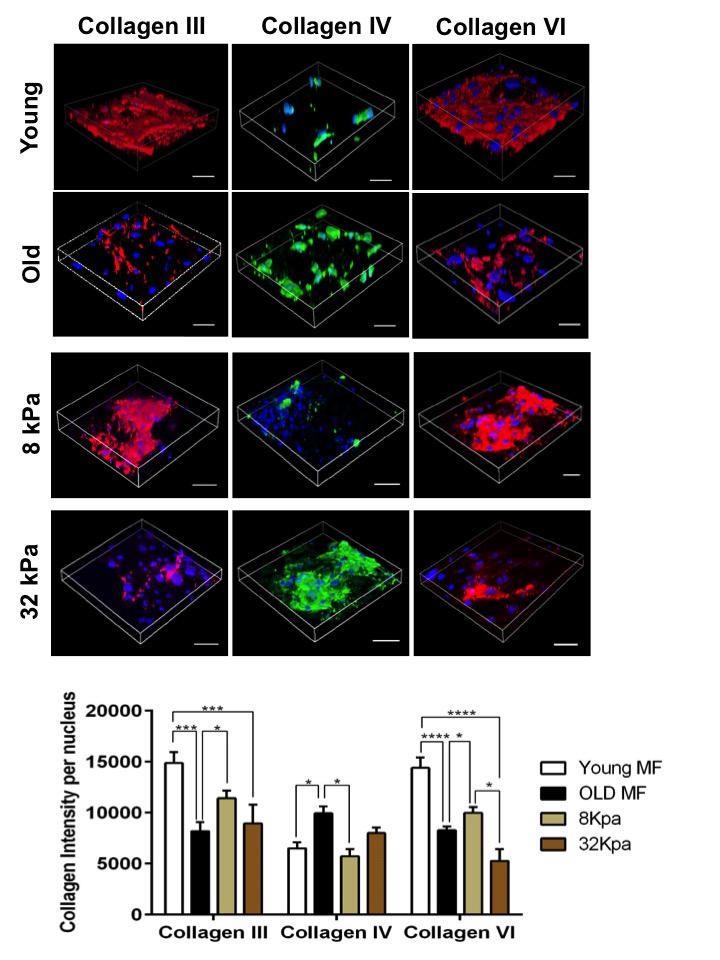
**

**Supplemental Figure 7: Determination of Latrunculin A and Leptomycin B dose response.**

Assessment of different dosages of Latrunculin A revealed that 0.1 uM was adequate to significantly reduce nuclear expression of YAP/TAZ (p<0.01). Conversely, assessment of different dosages of Leptomycin B revealed that 10ng was the optimal does required to significantly increase nuclear YAP/TAZ expression (p<0.01).


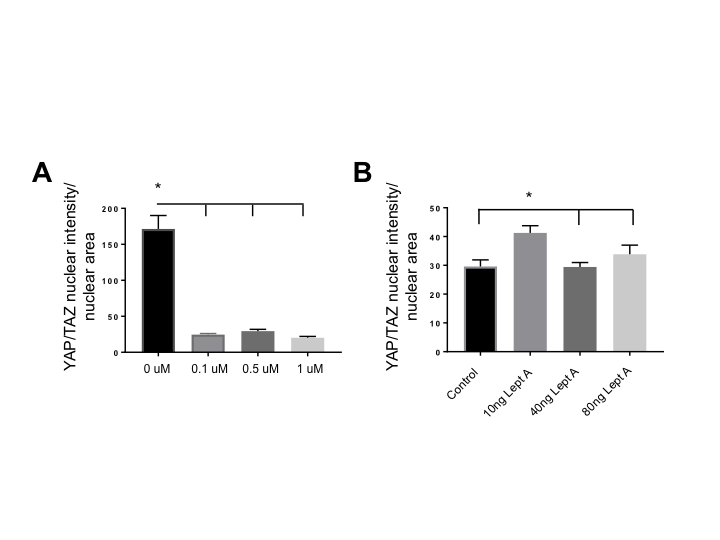

Supplement: Supplementary file 1 — Fig. S1 The histomorphometric analysis of collagen type III and elastin in young and old skeletal muscle. Fig. S2 DAPI and Tcf4 staining of fibroblasts isolated from the skeletal muscle of young and old mice. Fig. S3 The microarray gene expression profiling in young and old fibroblasts. Fig. S4 The expression of Pax7 & MyoD in the human muscle stem cells utilized in cell seeding experiments. Fig. S5 The resulting expression of desmin (A) and Tcf4 (B) from MuSCs seeded onto young and old decellularized and solubilized matrices. Fig. S6 The analysis of collagen composition between ECM deposited by young and old fibroblasts. Fig. S7 The dose response of latrunculin A (A) and leptomycin B (B). [file ACEL-16-518-s002.docx]
