# Supplementary figures and images for: Aging of the skeletal muscle extracellular matrix drives a stem cell fibrogenic conversion
Source: Aging Cell. 2017 Mar 30;16(3):518–28. doi: 10.1111/acel.12578 (PMC5418187; doi:10.1111/acel.12578)

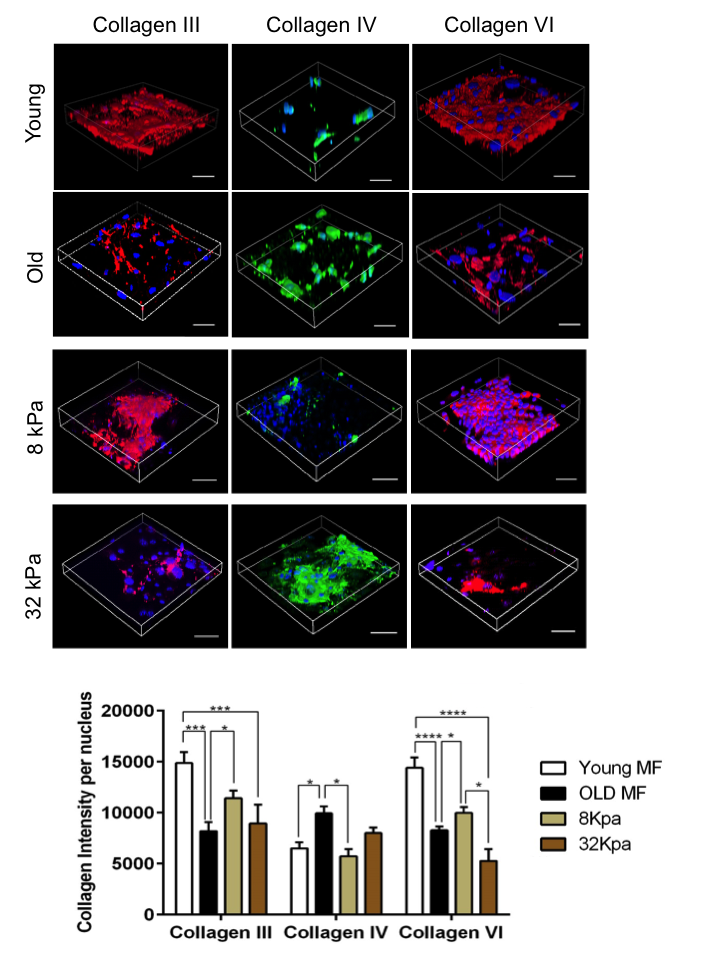

Supplement: Supplementary file 2 [file ACEL-16-518-s001.tif]
